# Evaluation of the Cutting Seton Technique in Treating High Anal Fistula

**DOI:** 10.7759/cureus.47967

**Published:** 2023-10-30

**Authors:** Asim M Almughamsi, Mohamed Khaled S Zaky, Abdullatif M Alshanqiti, Ibrahim S Alsaedi, Hamed I Hamed, Tariq E Alharbi, Ali A Elian

**Affiliations:** 1 General Surgery, Taibah University, Madinah, SAU; 2 Medicine, Taibah University, Madinah, SAU; 3 Medicine, Near East University, Lefkosa, CYP

**Keywords:** cured, recurrence, incontinence, cutting seton, high anal fistula

## Abstract

Objectives

Fistula-in-ano is a common condition that negatively affects the quality of life of its sufferers. A high anal fistula poses a significant challenge for surgeons due to its proximity to the anorectal ring and the potential risk of incontinence rather than recurrence. Many modalities have been used in a justified search for a satisfactory cure for the condition, but the seton remains a mainstay of surgical treatment. Therefore, the rationale of this study is to assess the outcome of treating a high anal fistula using the cutting seton technique in a hospital in Al Madinah, Saudi Arabia. The evaluation is intended through a retrospective analysis of patients' outcomes, comparing its effectiveness to similar articles.

Methods

This is a retrospective study that includes 50 patients with high anal fistulas who were treated with a cutting seton at the National Guard Hospital over a four-year period (2019-2022). Information obtained from medical records included clinical and demographic data. The data collected during the study was compiled and statistically analyzed using the SPSS Statistics version 26.0 (IBM Corp. Released 2019. IBM SPSS Statistics for Windows, Version 26.0. Armonk, NY: IBM Corp.). A p-value of <0.05 was considered statistically significant.

Results

A total of 50 patients with high anal fistula treated with a cutting seton were included: 82% were males and 18% were females, with 66% below 45 years of age. Approximately 92% had inter-sphincteric fistulas, and only 28% had a recurrent fistula. Almost all patients (98%) had an MRI done before surgery. Around 70% of patients were completely cured, 26% had minor complications, 8% of the operated patients experienced mild incontinence, and only one recurrence (2%).

Conclusion

The cutting seton is still a valid modality in treating patients with a high anal fistula, as it is considerably safe, effective, and yields good outcomes. Standard preoperative assessment and thorough surgical techniques are cornerstones for achieving a satisfactory outcome.

## Introduction

An anal fistula is a common surgical problem, especially among young men, with a male-to-female ratio of 2:1 and an estimated incidence of 1.2 to 2.8 per 10,000 in some Western populations and a prevalence of 0.2% [1]. An anal fistula is an abnormal route linking an internal opening within the anal canal to an external opening at the perianal skin. The usual presentation of patients with an anal fistula includes intermittent pain, itching, and the discharge of pus, feces, or blood [1].

Anal fistulae can be classified into low and high anal fistulae. In low fistulae, the internal orifice of the fistula begins distal to the puborectalis muscle, and the track usually passes across few or no sphincter muscle fibers and is relatively close to the surrounding skin. In contrast, in high anal fistulae, the internal orifice begins proximal to a significant number of muscle fibers; its route could be more complicated and not close to the skin [2].

Surgical treatment of a high anal fistula is not a simple surgical procedure due to the high risk of post-operative incontinence resulting from the affection of the external sphincter [3]. On the other hand, low anal fistula can be reliably treated only by fistulotomy [4]. The seton technique is one of the sphincter-sparing techniques applied in the treatment of high anal fistulas. The seton can be made of different types of materials to be inserted into the fistulous track for its elimination. Cutting, loose, and chemical setons are widely used to minimize the risk of anal incontinence [5]. Studies suggested that the cutting seton technique can be curative management for high anal fistula, with a low rate of incontinence and recurrence rates ranging from 8% to 22%. Furthermore, the loose seton technique can improve drainage and accelerate the stages of healing without a high risk of injury to the sphincter. Although the cutting seton can totally cure the fistula by gradually transecting the external sphincter muscle, it often results in some complications, including incontinence and even severe post-operative pain [6].

To the best of our knowledge, the management of high perianal fistula is controversial, and various treatment modalities have been tried. The seton is commonly practiced by many colorectal surgeons in the treatment of high anal fistula and is considerably associated with an acceptable outcome in treatment. Therefore, our rationale for this observational study is to assess the outcome of treating a high anal fistula using the cutting seton technique as a treatment modality used in Al Madinah, Saudi Arabia.

## Materials and methods

An observational study was conducted on 50 patients at the National Guard Hospital who had undergone surgical treatment for high perianal fistulas between 2019 and 2022. Data were retrieved from the medical records in the Surgery Department at the National Guard Hospital, including information on the anatomy of the fistula (de novo or recurrent), duration of seton placement, the material used, treatment outcome, and duration of follow-up. The study took place in Al Madinah, Saudi Arabia, from November 2021 to May 2022 and included all patients with confirmed high anal fistulas who were treated with the cutting seton technique.

Inclusion criteria encompassed both male and female patients with high anal fistula treated with the cutting seton technique from 2019 to 2022. Exclusion criteria were applied to patients who did not meet the aforementioned criteria. The variables included gender, age, fistula duration, fistula anatomy (whether de novo or recurrent), duration of seton placement, materials used, follow-up duration, and treatment results.

The data were statistically described in frequencies and valid percentages for categorical variables. The Chi-square or Fisher's exact test was performed to analyze relationships among categorical variables. A p-value less than 0.05 was considered statistically significant. All statistical calculations were conducted using SPSS Statistics version 26 (IBM Corp. Released 2019. IBM SPSS Statistics for Windows, Version 26.0. Armonk, NY: IBM Corp.). Qualitative data were presented as numbers and percentages for categorical variables, while quantitative data were presented as means and standard deviations for continuous variables.

This retrospective study was performed following the tenets of the Declaration of Helsinki and was approved by the Review Committee of the Faculty of Medicine at Taibah University, Al Madinah, Saudi Arabia (study ID: STU-21-006\date of decision: 5/02/2022) registered at the US Department of Health and Human Services (IORG0008716-IRB00010413).

## Results

Included in the study were a total of 50 patients treated with a cutting seton for anal fistula. The majority of these patients were under the age of 45 (66%), male (82.0%), had the inter-sphincteric perianal fistula type (92%), and presented with new anal fistulas (72%). Nearly all patients (98%) underwent MRI prior to the surgical procedure. Complete characteristics are provided in Table 1.

**Table 1:** Characteristics of the patients

Parameters	Category	Count (n=50)	Percentage
Age	≤45	33	66
	>45	17	34
Gender	Female	9	18
	Male	41	82
Type of fistula	Inter-sphincteric perianal fistula	46	92
	Trans-sphincteric fistula	4	8
State of fistula	New	36	72
	Recurrent	14	28

**Figure 1:**
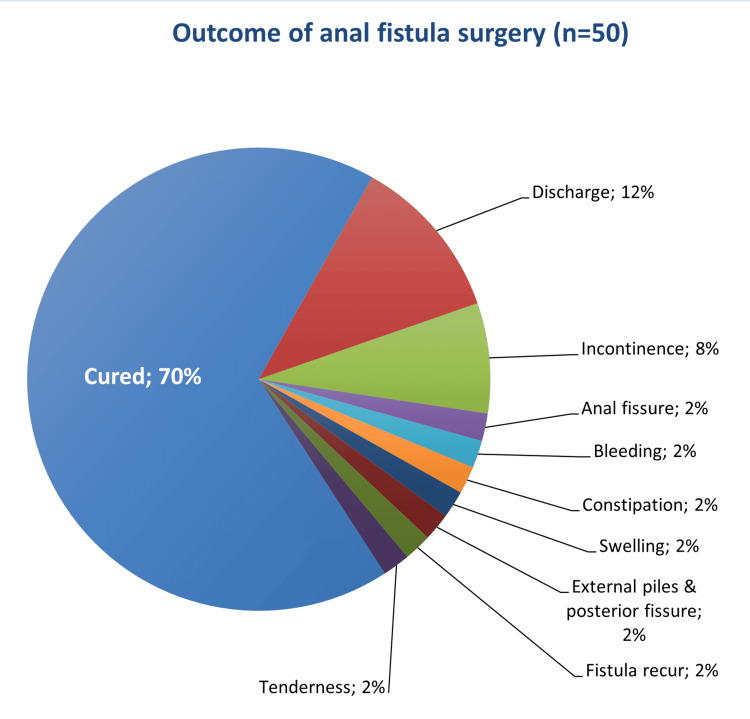
Outcome of anal fistula surgery

**Figure 2:**
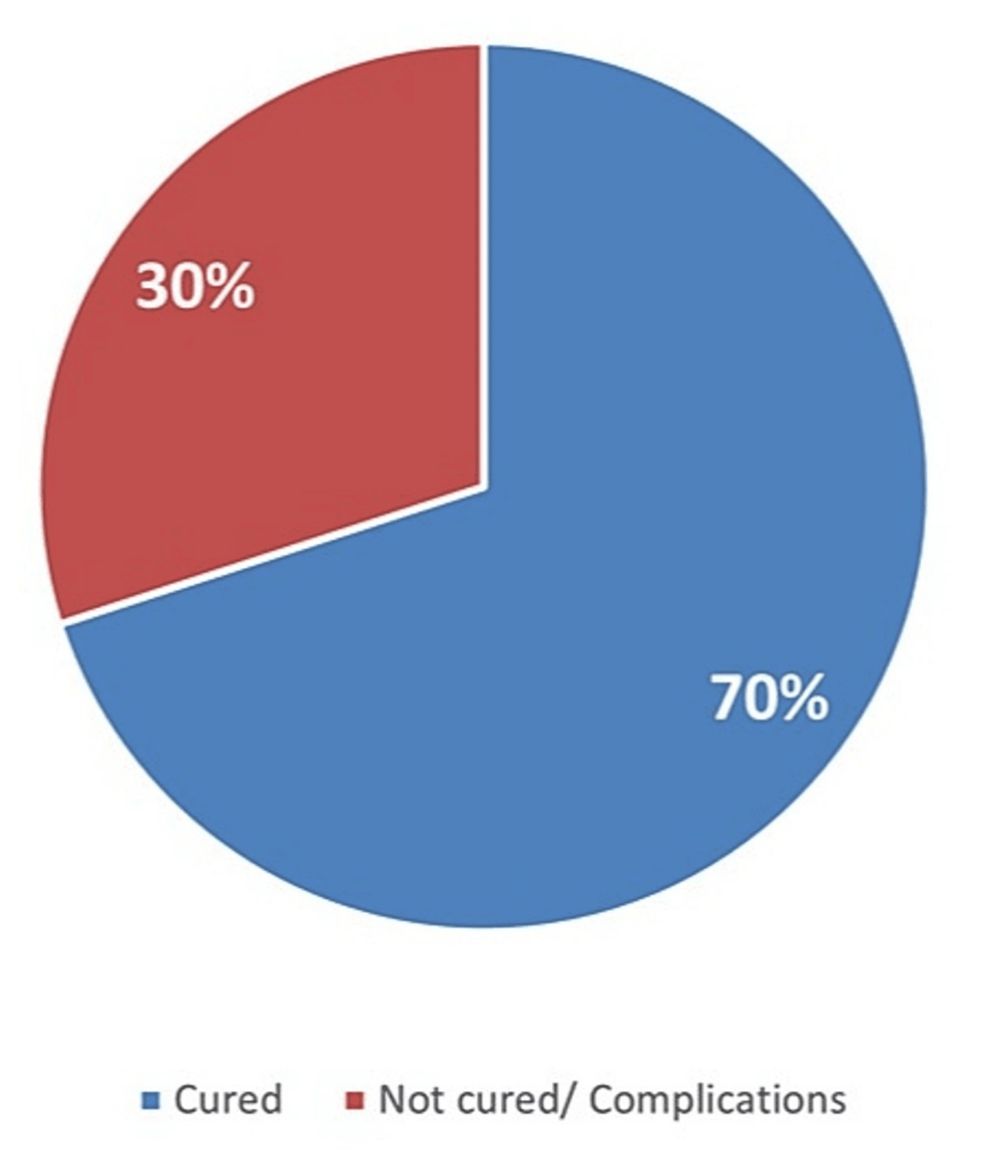
Cure rate of anal fistula surgery (n=50)

**Figure 3:**
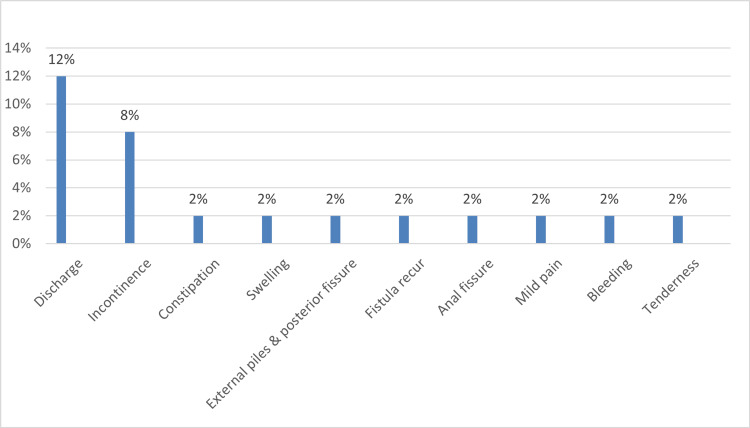
Complications of anal fistula surgery

Table 2 outlines the characteristics of anal fistula operations. Approximately two-thirds of the total patients experienced an uneventful outcome, while the remaining exhibited post-operative complications, including discharge (12%) and varying degrees of incontinence (8%) (Figures 1-3). Moreover, 84.4% of the patients did not require an additional posterior extrauterine adhesion (PEUA) or fistulotomy, and about 79.1% necessitated a second surgery for seton removal. The majority of patients were followed up for over three months (60%).

**Table 2:** Characteristics of pre- and post-operative anal fistula PEUA: posterior extrauterine adhesion, MRI: body mass index

Parameters	Category	Count (n=50)	Percentage
Preoperative investigation	MRI	49	98
	MRI and colonoscopy	1	2
Operation details	Fistulotomy and PEUA	50	100
Outcome of surgery	Cured	35	70
	Anal fissure	1	2
	Complicated by bleeding	1	2
	Constipation	1	2
	Swelling	1	2
	External piles and posterior fissures	1	2
	Fistula recur	1	2
	Incontinence	4	8
	Mild pain	1	2
	Discharge	6	12
	Perianal tenderness	1	2
Requiring second surgery for removing seton	Yes	12	24
	No	35	74.5
The duration between the first and second surgery	<5 months	7	58.3
	≥5 months	5	41.7
Requiring another PEUA and fistulotomy	Yes	3	13.6
	No	19	86.4
Duration of follow-up	≥3 months	30	60
	<3 months	20	40

Regarding the factors associated with the need for a second surgery to remove the seton, only gender exhibited a statistically significant impact. Male patients displayed a lower requirement for a second surgery compared to females. Comprehensive details are presented in Table 3.

**Table 3:** Comparing the magnitude of requiring a second surgery for removing seton among the different subgroups

Subgroups		Require second surgery for removing seton			p-value
		Yes		No	
Gender	Female	4 (57.1)	3 (42.9)		0.026
	Male	5 (13.9)	31 (86.1)		
Age	≤45	5 (16.7)	25 (83.3)		0.417
	>45	4 (30.8)	9 (69.2)		
Type of fistula	Inter-sphincteric perianal fistula	8 (20.5)	31 (79.5)		1.000
	Trans-sphincteric fistula	1 (25)	3 (75)		
State of fistula	New	5 (15.6)	27 (84.4)		0.201
	Recurrent	4 (36.4)	7 (63.6)		
Duration of follow-up	≥3 Months	5 (20)	20 (80)		1.000
	<3 Months	4 (22.2)	14 (77.8)		

## Discussion

Anal fistula is a complex health issue, causing concerns for both patients and surgeons. The apprehensions arise from the intricate pathophysiology, the risk of incontinence, and the elevated recurrence rates, particularly in complex cases [7].

High perianal fistula procedures are regarded as one of the most critical challenges for many surgeons. They often entail a high recurrence rate and various complications, such as anal incontinence. The success of these procedures heavily relies on the surgeon's skills, experience, and the complexity of the fistula. Several techniques can be employed to treat high fistulas, with cutting seton playing a pivotal role in such surgeries. Several studies investigated the efficacy of non-cutting seton techniques in high anal fistula management. A randomized controlled trial published by Jones et al. (2016) compared non-cutting seton with cutting seton in the treatment of high anal fistulas. The study demonstrated comparable success rates between the two groups, with non-cutting seton showing a lower risk of fecal incontinence. These findings suggested that non-cutting seton techniques can be an effective treatment option for high anal fistulas while mitigating the risk of adverse functional outcomes [8]. This study aims to evaluate the outcomes of surgical interventions using the cutting seton technique in managing high anal fistulas at a hospital in Al-Madinah.

The prevalence of anal fistulas is typically reported to be highest during the third and fourth decades of human life worldwide. In our study, 66% of the patients were aged 45 years or younger, consistent with another study in Saudi Arabia that reported a mean age of 39.5 years [2]. Our data also indicates a higher representation of males than females (82% and 18%, respectively). These findings align with previous studies; Alkhawaga et al. (2019) reported that males comprised two-thirds of the total patients [9], and Shirah et al. (2020) found that 80.1% of the patients were male and 19.9% were female. Tarasconi et al. (2021) also noted that adult males with abscesses and/or fistulas were twice as common as affected females [10]. However, our results differ from an Egyptian study that reported a higher prevalence of perianal fistulas in females than males [11].

Efficient preoperative evaluation is essential for accurately diagnosing the type of fistula and understanding its anatomy. In our study, nearly all patients underwent preoperative evaluation with an MRI fistulogram, which is sensitive and crucial for determining the type of fistula and identifying any side branches. This practice aligns with Morsi's (2019), where an MRI fistulogram was used as a diagnostic tool for all cases [12].

The study revealed that inter-sphincteric perianal fistula was the predominant type of fistula. This finding contrasts with Alkhawaga et al. (2019), who reported that inte-sphincteric perianal fistula was diagnosed in only 24% of patients. Additionally, inter-sphincteric fistula was observed in about one-third of the patients in a study conducted in Brazil [13]. The cutting seton is an age-old surgical technique still widely employed to manage complex fistula cases. It is a relatively simple method with an acceptable and appropriate cure rate [14]. In our current study, 70% of high anal fistula patients were treated with cutting seton without developing complications after surgery, and 74.5% did not require a second surgery for seton removal. These results are consistent with those of a study in Egypt, which indicated that 74% of total anal fistula cases were successfully cured without complications [4].

In this study, postoperative complications included discharge (12%), incontinence (8%), fissure, bleeding, constipation, swelling, external piles, fistula recurrence, and perianal tenderness (2%). Similarly, Morsi (2019) indicated that discharge was the most common complication observed in all his cases (100%), with approximately 8% of cases reporting incontinence. However, Shawki and Wexner (2011) [15], as well as Alkhawaga et al. (2019), reported that the most prevalent postoperative complication of fistula surgery is sepsis (15% and 16%, respectively), which differs from our findings. Worldwide, the incontinence rate after anal fistula surgical management using various types of medical materials for the seton varies from 0% to 62%. Additionally, the recurrence rate is reported to range from 2.4% to 16% [2,16], which aligns with our study's findings. Similar results were also reported by a Saudi study, which showed a 2.4% rate of fistula-in-ano recurrence [2]. However, Izadpanah et al. (2016) stated that no patients in their study complained of incontinence, which differs from our findings [17].

In addition, when comparing factors such as gender, age, type of fistula, fistula state, and the duration of follow-up with surgical outcomes, no significant effects on the cure rate were observed. In our study, males achieved a complete cure more frequently than females (75.6% and 44.4%, respectively). These findings align with Hyman et al. (2009), who reported that females were significantly correlated with a higher risk of wound healing failure after surgery [18]. However, other authors found no significant difference between males and females in terms of fistula recurrence [19]. Moreover, Garcia-Aguliar et al. (1996) reported that females were more strongly associated with the risk of fecal incontinence after surgery for anal fistula than males [20]. In our present study, being older than 45 years, having a new fistula diagnosis, and having a trans-sphincteric fistula were associated with a higher cure rate when compared to younger patients.

A notable strength of our study is that it not only evaluated the efficacy of surgical intervention using the cutting seton for managing high anal fistulas but also performed a comparative analysis, examining how factors such as gender, age, fistula type, and fistula condition influence the outcomes of cutting seton surgery. Nevertheless, there are limitations to this study, primarily stemming from its reliance on data from a single hospital with a relatively small sample size. Additionally, being observational in nature, it focused exclusively on the outcomes of cutting seton surgery and did not explore other operative interventions. In light of these limitations, it is recommended for future research to encompass larger sample sizes and include various types of anal fistula surgeries for a more comprehensive understanding of outcomes and factors influencing them.

## Conclusions

The utilization of the cutting seton technique in treating patients with high anal fistulas is highly safe and effective, with consistently good results. When observing this surgical method used for treating high anal fistulas, cutting seton demonstrates low rates of recurrence and incontinence. The exploration of the inter-sphincteric plane not only prevents infection but also clearly delineates high extensions, allowing for effective management and reducing the risk of recurrence. In our study, the cure rate was 70% among patients with high anal fistula, and only 8% reported complaints of incontinence.
